# Axillary uniportal video-assisted thoracoscopic surgery for bullae is a cosmetically superior approach to primary spontaneous pneumothorax: a case report

**DOI:** 10.1186/s13019-021-01703-8

**Published:** 2021-10-26

**Authors:** Weijiang Ma, Xiuping Deng, Ming Wen, Limin Yang, Xun Ouyang, Xin Liu, Yin Liu

**Affiliations:** 1 Department of Thoracic Surgery, Second Affiliated Hospital of Kunming Medical University, NO.374th Dianmian Road, Kunming, 650101 Yunnan Province China; 2 Department of Thoracic Surgery, Honghe Prefecture Third People’s Hospital, Gejiu, 661000 Yunnan Province China; 3 Department of Emergency Trauma Surgery, Second Affiliated Hospital of Kunming Medical University, Kunming, 650101 Yunnan Province China; 4 Department of Plastic Surgery, Second Affiliated Hospital of Kunming Medical University, NO.374th Dianmian Road, Kunming, 650101 Yunnan Province China

**Keywords:** Primary spontaneous pneumothorax, Bulla, Video-assisted thoracoscopic surgery (VATS), Bullectomy, Case report

## Abstract

**Background:**

Bulla is a common cause of primary spontaneous pneumothorax. Video-assisted thoracoscopic surgery (VATS) through the lateral chest wall is a common surgical approach and an effective treatment for this condition, but postoperative incision scars affect the aesthetic outcome. VATS via axillary approach can hide the scar in the axilla, and the wound in its natural state is invisible; this greatly improves the cosmetic appearance. To our knowledge, this is the first report of VATS-based bullectomy via the axillary approach in a patient with spontaneous pneumothorax.

**Case presentation:**

A 20-year-old female patient was admitted to the hospital with a 2-day history of chest tightness and chest pain. Plain chest computed tomography showed right spontaneous pneumothorax, lung compression of 75%, and right pulmonary bulla. After complete preoperative examination, VATS bullectomy via right axillary approach was performed. During the operation, a bulla measuring about 4 × 4 cm was found at the apex of the right lung and resected. The incision healed well, and the patient was discharged after surgery.

**Conclusions:**

VATS bullectomy via axillary approach is safe and feasible, with the incision hidden in the axilla and not visible in the natural state. This method leaves no scar on the chest wall and has good cosmetic outcome.

## Background

Pulmonary bullae are common causes of primary spontaneous pneumothorax. When the bullae rupture, air enters the pleural cavity from the lung, the negative pressure of the pleural cavity disappears, and the lung tissue is compressed and collapsed, resulting in chest tightness, chest pain, and dyspnea. With the development of medical devices and minimally invasive techniques, video-assisted thoracoscopic surgery (VATS) has been widely used to treat a variety of thoracic diseases including bullae [1]. To the best of our knowledge, VATS bullectomy via the axillary incision approach has not yet been reported.

## Case presentation

A 20-year-old female patient presented to our hospital with complaints of chest tightness and chest pain for 2 days. Physical examination revealed the following: temperature, 36.6 °C; heart rate, 92 beats/min; blood pressure, 100/65 mmHg; oxygen saturation, 88% (without oxygen inhalation); and body mass index (BMI), 20.93 kg/m^2^. Chest auscultation showed that the right respiratory sound was significantly weakened. Chest computed tomography (CT) showed right pneumothorax with 75% pulmonary compression and bullae in the upper lobe of the right lung. No obvious abnormalities were found in electrocardiography, blood routine and blood biochemistry tests, coagulation function, and other examinations. Accordingly, the patient was diagnosed as right primary spontaneous pneumothorax caused by rupture of pulmonary bulla, a VATS bullectomy was arranged. During the waiting period, the patient was given oxygen inhalation to alleviate symptoms, and thoracentesis was performed through the second intercostal space at the right mid-clavicular line. This resulted in elevation of oxygen saturation to 95%.

**Fig. 1:**
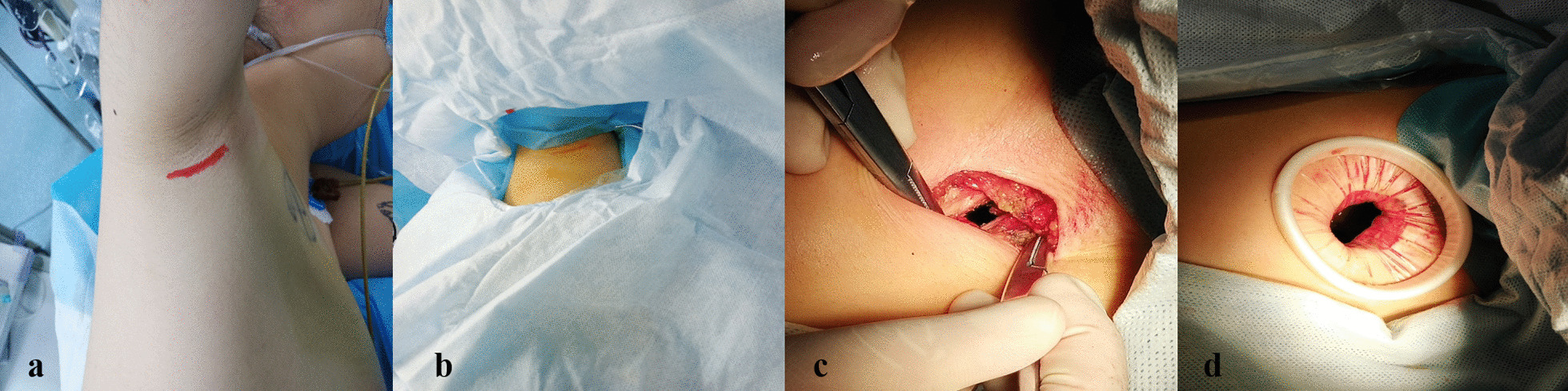
Choice of position and incision. **a** The upper limb was suspended in the lateral decubitus position. **b** Routine disinfection and placement of surgical towel. **c** Separated subcutaneous tissues and muscles. **d** Incision protector in place

**Fig. 2:**
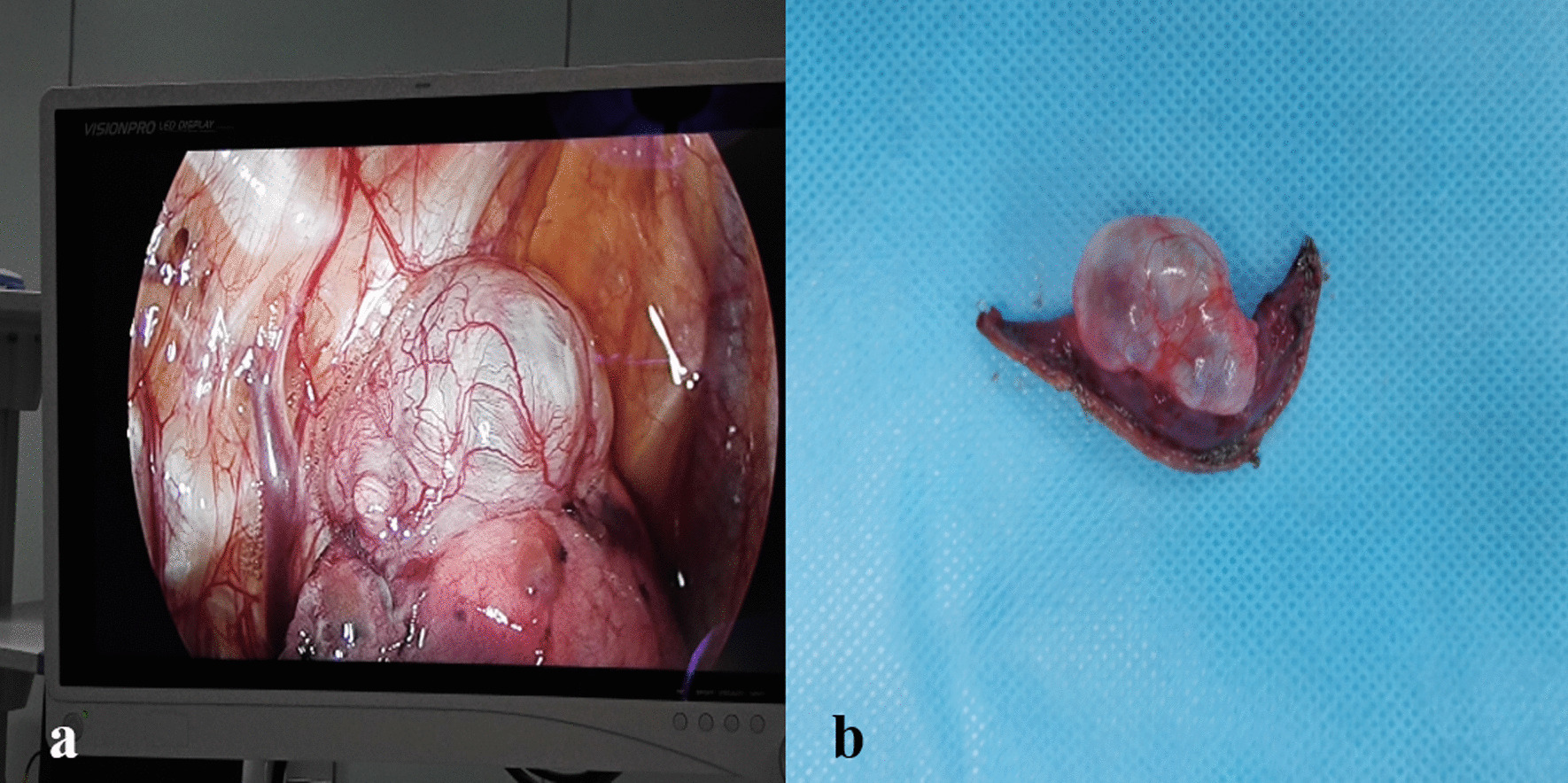
Location of the bulla. **a** A bulla measuring about 2 × 3 cm in size at the apex of the upper lobe of the right lung. **b** After resection with a linear cutting stapler

**Fig. 3:**
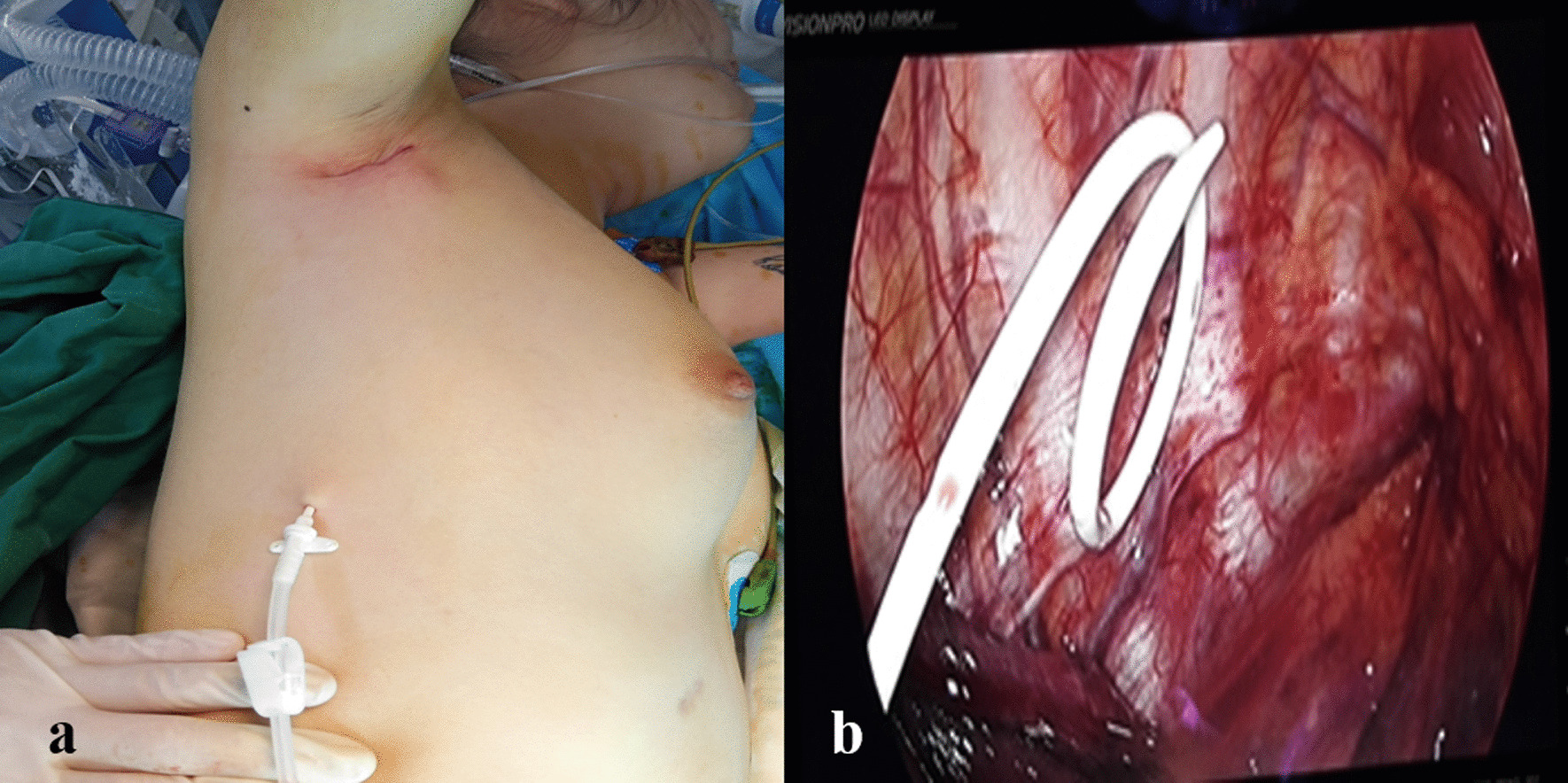
Thin drainage tube. **a** Outside view. **b** Inside view

**Fig. 4:**
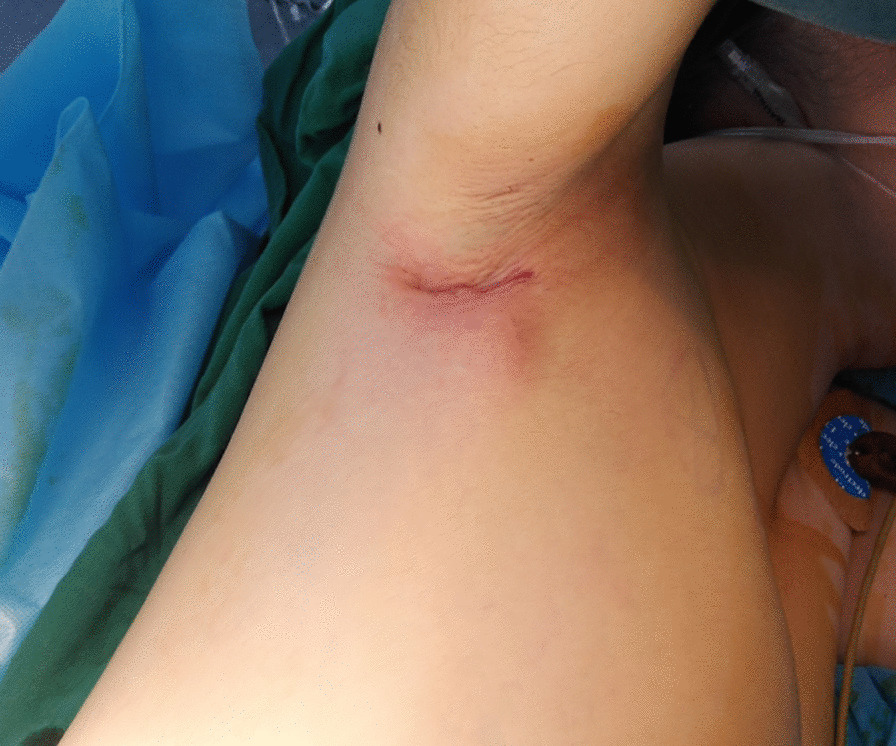
Incision after suture

**Fig. 5:**
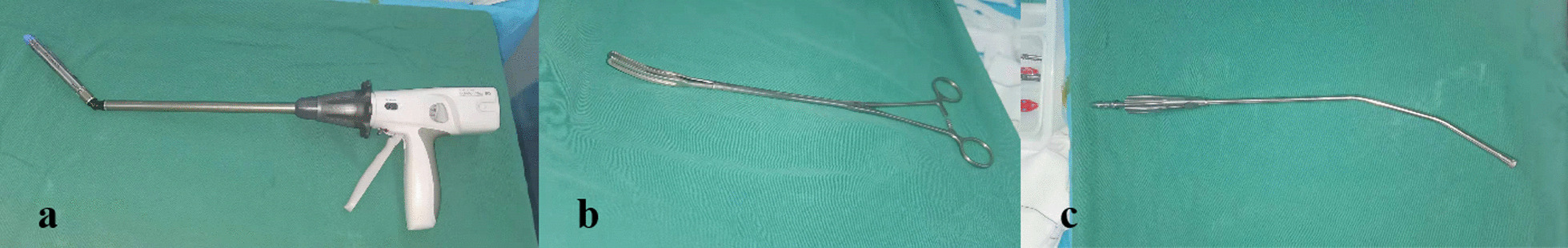
Special instruments used. **a** Double joint linear cutting stapler. **b** Double joint linear cutting stapler. **c** Bent aspirator

After general anesthetic induction, endotracheal intubation was performed via the routine double-lumen endotracheal intubation procedure. The patient was placed in a lateral decubitus position. The upper limb was suspended to fully expose the axilla. After routine disinfection, a 2.5-cm incision was made along the lower dermis of the right axillary fold. Subcutaneous tissues and muscles were separated layer by layer, and the thorax was entered through the second intercostal space; an incision protector was inserted (Fig. 1). During operation, pay attention to avoid damage the nerves in the axilla, the long thoracic nerve in particular. Exploring the thorax under thoracoscopy, we found a bulla measuring about 2 × 3 cm in size at the apex of the upper lobe of the right lung, and wedge resection was performed with a linear cutting stapler (Fig. 2). After the operation, a thin drainage tube was placed from the seventh intercostal space on the right midaxillary line with closed underwater seal drainage (Fig. 3). The chest was closed layer by layer, and the skin was sutured with 4–0 absorbable sutures (Fig. 4). Due to the high position of entering the thorax, it is difficult to operate, and the surgical instruments need higher flexibility, so we used some special instruments (Fig. 5). The postoperative management and pain control of this technique are similar to conventional, including oxygen inhalation, nebulization therapy, expectorant, analgesic treatment and cough training. The patient recovered well and was discharged on day 3 after surgery. One month later, a follow-up chest CT showed no abnormality. No related nerve injury was found during follow-up, such as "pterygoid shoulder".

## Discussion

VATS bullae resection has become the preferred procedure for the treatment of primary spontaneous pneumothorax. The recurrence rate is not significantly different than thoracotomy, but it has the advantages of less trauma, faster recovery after surgery, and shorter hospital stay [2, 3]. The choice of surgical approach for VATS is very important. When the surgical approach is not appropriate, it will cause visual field limitation, and increase the difficulty of surgery. The change of surgical approach for VATS also reflects the development of minimally invasive technology. The initial approach was thoracoscopic small incision, with an observation port at the seventh or eighth intercostal space in the midaxillary line and an incision of about 5 cm at the fourth or fifth intercostal space under the axilla as the operation port. Later, Professor Mckenna pioneered the designing and completion of the four-port VATS, marking the formal opening of thoracoscopic surgery [4]. With advancements in the minimally invasive techniques and the development of thoracoscopic instruments, the 3-ports VATS approach comprising an observation port, a main operation port, and a secondary operation port subsequently emerged. Later, on the basis of the 3-ports approach, the secondary operation port was cancelled, which led to the 2-ports approach. The advent of double-joint instruments and variable-direction cutting staplers laid the foundation for the advent of single-port VATS, with the thoracoscope and instruments accessible through the same port. These changes in the VATS approach reflect surgeons' efforts to reduce surgical trauma.

However, to date, regardless of the VATS approach, the incision is on the lateral chest wall, and the residual scar after surgery will still affect the aesthetics, which is always unsatisfactory. Therefore, we assume that if the incision is placed in the axilla and the arms drop naturally, the incision can be hidden in the axillary space, which will result in favorable cosmetic appearance. Considering the specific needs of the patient’s occupation and the high requirement of postoperative aesthetics, we tried to perform axillary uniportal VATS bullectomy after obtaining the patient’s written informed consent. Fortunately, the postoperative results were in line with expectations, and the incision could be concealed in the axilla. The patient had an uneventful recovery and was discharged smoothly. It is also worth noting that, due to the high position of entering the thorax, it is difficult to operate, therefore, the technique needs to be carefully selected after full evaluation. In my opinion, it only applies to resecte bullae at the apex of the lung. This case is the first step of our new idea, which highlights the feasibility for popularizing this method. However, we acknowledge that the specific effectiveness and safety still need to be verified in more cases through clinical trials.

## Conclusions

VATS bullectomy via axillary approach is safe and feasible, with the incision hidden in the axilla and not visible in the natural state. This method leaves no scar on the chest wall and has good cosmetic outcome.

## Data Availability

The datasets used and/or analyzed in the current article are available from the corresponding author on reasonable request.

## Abbreviations

VATS: Video-assisted thoracoscopic surgery
BMI: Body mass index
CT: Computed tomography
BMI: Body mass index
